# Compression Molding of Low-Density Polyethylene Matrix/Glass-Fiber-Reinforced Thick Laminates

**DOI:** 10.3390/polym16192722

**Published:** 2024-09-26

**Authors:** Fabrizio Quadrini, Giorgio Patrizii, Alice Proietti, Leandro Iorio, Denise Bellisario, Loredana Santo

**Affiliations:** Department of Industrial Engineering, University of Rome “Tor Vergata”, Via del Politecnico 1, 00133 Rome, Italy; giorgio.patrizii@uniroma2.it (G.P.); alice.proietti@uniroma2.it (A.P.); leandro.iorio@uniroma2.it (L.I.); denise.bellisario@uniroma2.it (D.B.); loredana.santo@uniroma2.it (L.S.)

**Keywords:** thermoplastic composites, glass fabrics, fiberglass, low density polyethylene, compression molding, recycling, repairing

## Abstract

Thermoplastic fiberglass was compression molded in the form of thick panels with a nominal thickness of 10 mm and a size of 300 × 300 mm^2^. A simplified procedure was adopted to speed up the lamination procedure and adapt it to the aim of recycling waste, glass fibers, fabrics, and thermoplastic films. Low density polyethylene was used as a matrix to simplify the laboratory process, but the same procedure can be extended to other thermoplastic film, such as polyamide. The final thermoplastic composite shows unique properties in terms of its repairability, and its performance was improved by increasing the number of repairing repetitions. For this aim, a repairability test was designed in the bending configuration, and three consecutive cycles of bending/repairing/bending were carried out. The static mechanical properties of the final thermoplastic composite were, conversely, low in comparison with traditional fiberglass because of the choice of a polyethylene matrix. The bending tests showed that the maximum strength was lower than 10 MPa and the elastic modulus was less than 1 GPa. Nevertheless, the toughness of the thermoplastic composite was high, and the samples continued to deform under bending without splitting into two halves.

## 1. Introduction

This study combines two aspects of recycling: the first is that recycling needs easy procedures which do not affect the performances of the recycled products while the second is that low-density polyethylene (LDPE) is often recycled because of its popularity as a packaging material and its easy reprocessability. Conversely, recycled materials with a high added value, such as fiberglass, have more potential to re-enter the market. Fiberglass is a common material that is widely used in many applications; generally, it is a thermoset composite with a polyester matrix and continuous glass fibers (GFs) in different configurations, from fabrics to mats and unidirectional tapes. Recently, continuous glass fibers were used in thermoplastic matrices, to take advantage of the behavior of this class of materials in terms of their toughness and easy processing. For this aim, LDPE was also evaluated, due to its availability on the market and the low cost of both virgin and recycled material. Processing LDPE in high production runs is easy, because of its low viscosity after molding and its relatively low processing temperature, in comparison with other thermoplastics. In 2021, Ravichandran et al. used carbon, Kevlar, and glass fibers as reinforcements to create LDPE matrix composites through compression molding [1]. Their study examined the physical, mechanical, and thermal characteristics of the molded composites to determine the influence of the different fibers on their properties. They observed that the most noticeable effects resulted from carbon fibers with regard to mechanical performance, whereas Kevlar fibers enhanced water absorption. 

Nevertheless, they concluded that these composites are viable for use in various engineering sectors, like automotive and construction.

**Figure 1:**
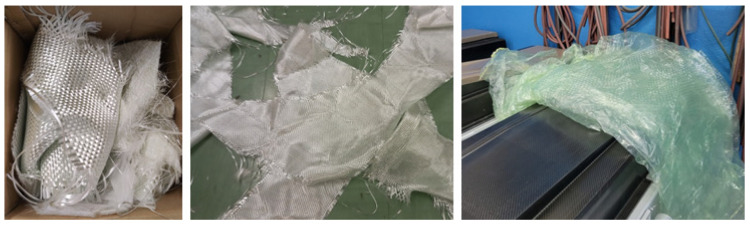
Typical waste from composite manufacturers: residuals of dry glass-fiber fabrics and used thermoplastic films.

In 2012, Jahan et al. proposed a work in which there was a comparison between jute-fiber-reinforced and glass-fiber-reinforced LDPE composites [2]. Both were made by using compression molding at a temperature of 120 °C. Many physical and mechanical properties were compared, and the results showed that the glass-reinforced composites had better mechanical stability compared to the jute composites. This kind of study, in which LDPE is presented together with natural-based materials, is typical, as LDPE can be a sustainable material source as well, when sourced from recycling. In general, glass fibers and thermoplastic films are common waste products from industries (Figure 1). Jute is biodegradable, but the LDPE matrix and glass fibers are not, and, therefore, several waste management and recycling issues may arise. Regarding this topic, in 2024, Soo et al. proposed the use of bio-based PE, derived from renewable resources like corn and sugarcane, which represents an eco-friendly alternative to LDPE [3]. The production of bio-PE composites is growing, due to environmental issues and regulation pressures. Furthermore, the PE bio-composites employ agricultural waste as filler materials, representing a sustainable and environmentally friendly alternative to traditional plastics. They have interesting potential applications in the automotive, furniture, and building construction industries, if sufficient performance and durability criteria can be reached.

In 2022, Karem et al. proposed a study of the mechanical characterization of LDPE as a matrix in composite materials with glass fibers [4]. In this work, composites were produced with different fractions of glass fibers (up to 22 wt%) by hand lay-up, and their mechanical performances were tested under impact, tensile, and bending tests. As expected, by increasing the glass fiber content, mechanical properties rose. Similar results had been already obtained by other researchers with discrete glass reinforcements. Chimeni et al., in 2018, used glass beads incorporated into LDPE [5]. Smaller particle sizes had a greater impact on the composite behavior under several loading conditions because of their increased specific surface area. In general, a remarkable distinction was noted between virgin and recycled LDPE because of the contamination found in the recycled material. Falcone et al., in 2018, used hollow glass microspheres through melt blending [6]. The improvement of the interaction between fillers and the LDPE matrix was achieved following their surface modification with a proper organo-silane.

In 2021, Gulati et al. discussed the injection molding of hybrid composites with linear-LDPE (LLDPE) reinforced with date pits and glass fibers [7]. The highest tensile strength achieved was 20.2 ± 0.8 MPa, with a reinforcement composed of 10% date pit and 20% glass fiber. Higher percentages of glass fiber were discovered to improve the water-resistant characteristics. These performances are typical for LDPE-matrix composites, which are characterized by high ductility and toughness but low strength, in comparison with other thermoplastics. For this reason, hybrid composites are often presented by researchers, mixing different typologies of fibers and particles. In 2017, Azizi et al. studied LDPE-matrix composites with a graphene-like material that was prepared by using a melt-intercalation compounding method [8]. The composites were molded by compression molding, and a minimal decrease in both the melting point and the crystallinity compared to the pure polymer were found. Recently, Krehula et al. studied, in 2024, the incorporation of hematite particles into LDPE, and they discussed the positive effects on the mechanical properties of the composites [9].

The easiness of particle incorporation into LDPE melts has always pushed researchers to find new composite mixtures, mainly by using natural feedstocks. In 2022, Sakdapipanich et al. proposed a composite that was obtained by incorporating macca carbon (MC) powder, a biomass derived from macadamia nut cultivation that emits far-infrared radiation, into a matrix of low-density polyethylene (LDPE) [10]. It had potential to be used for antimicrobial packaging applications. The results showed that incorporating MC powder into the LDPE/MC film composite at a proportion of 0.5% (by weight) caused the maximum percentage of microorganism decrease. In 2023, a study by Ferrandez et al. used LDPE waste as secondary raw material in the production of new, eco-friendly gypsum composites [11]. In 2023, Melikoglu et al. proposed innovative bio-composites for packaging films by inserting cellulose and cellulose nanocrystals, obtained from safflower head waste, into LDPE [12]. In the same year, Rodriguez-Fabia et al. used cellulose nanofibrils as fillers in LDPE bio-composites [13], whereas Sharma et al. produced nanocomposites by adding BiOCl nanoparticles for UV-shielding applications [14], and Yucesoy et al. used magnesium hydroxide and magnesium carbonate at various weight ratios to create flame-retardant LDPE composites [15].

The scientific literature shows that LDPE has a tradition of being an eco-friendly material, despite its synthetic nature. Moreover, studying LDPE composites reveals that recycling is always an option, and the best advantages for the environment are achieved when both the matrices and the fillers are recycled or are recovered from waste. Gulser et al., in 2020, combined metal waste from the iron-steel industry with plastic waste from the plastic industry, with the goal of minimizing the overall waste from industrial production processes and promoting sustainable waste management [16]. Different quantities of blast furnace dust, which represents a major waste material from the iron–steel industry, was mixed with LDPE with the effect of enhancing the heat resistance, the thermal conductivity, and the wear resistance of these composites.

**Figure 2:**
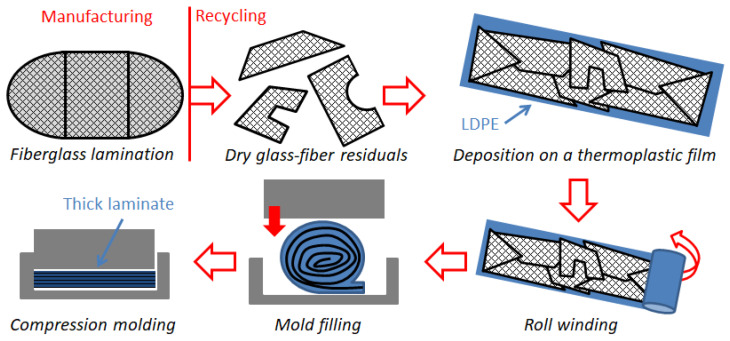
Lamination procedure to mold thick thermoplastic laminates from waste dry fabrics.

In this line of eco-friendly composite production, the idea at the foundation of the current study is introducing a simple manufacturing process for LDPE composites by integrating different industrial waste streams. Process simplicity is a mandate to guarantee its success in the industrial sphere. For this study, waste dry fabrics were collected from factories that produce fiberglass products, and these were processed with LDPE films that were used for packaging. In order to simplify its supply, the adopted LDPE film was virgin, but it is a typical material that, in future, could be recovered as waste. Thick thermoplastic laminates were compression-molded after a simplified lamination procedure (Figure 2). The final composites showed good agglomeration and exhibited unique properties in terms of their repairability.

## 2. Materials and Methods

Dry glass-fiber fabrics were recovered from fiberglass manufacturers in the form of large residuals after fabric cutting. These materials have the same properties of virgin fabrics but are not usable because of the shape. For the matrix, an LDPE roll with a thickness of 60 μm and a width of 300 mm was acquired. LDPE roll is a typical packaging material and can be recovered by recycling streams, as the glass-fiber fabrics were. However, in this study, a virgin roll was used to simplify the laboratory procedure and to reduce possible uncertainties arising from the use of recycled LDPE. In following research activities, thanks to the outcomes of this study, the manufacturing procedure was successfully extended to 100% recycled composites and different thermoplastic matrices, as partially discussed in Section 5. This approach was successfully applied to other waste composite materials also, in the past [17,18].

**Figure 3:**
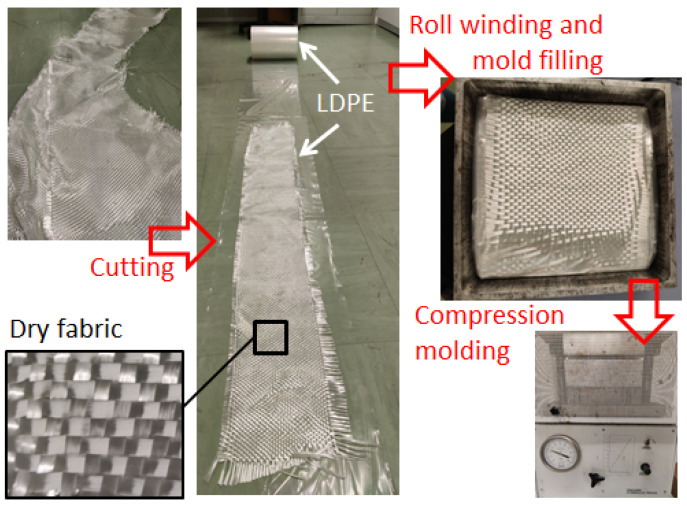
Manufacturing steps for the thermoplastic thick laminate by recycling the residuals of dry glass-fiber fabrics.

The first aspect to define for a successful industrial process is the clarity and easiness of the fabrication steps, especially in the case of recycling processes. For composites, the main issues are related to the difficulty of managing large raw materials (fabrics, sheets) and the need to implement a reliable lamination procedure. Dealing with residuals from composite manufacturing (parts of fabrics and mats, waste packaging, or vacuum bagging films) is challenging as, most of the time, they are crumpled during manufacturing and are very difficult to re-use subsequently. Therefore, for recycling, an easy lamination procedure must be carried out in parallel with the manufacturing of the composite part, to recycle the residuals and the waste materials without impacting the main industrial process. The proposed process is shown in Figure 2. The dry fabrics were deposited on thermoplastic sheets and wound into rolls to make molding preforms. These preforms were compression-molded to make the laminates. Because of the combined action of the mold temperature and the punch pressure in the press, the thermoplastic film melts, and the molten polymer infiltrates the dry fabrics. In this study, the procedure of Figure 2 was applied to make thick thermoplastic composite laminates in the laboratory. In Figure 3, some details of the production phase are shown.

The largest residuals of glass-fiber fabrics, collected from a fiberglass manufacturer, were selected and cut to the width of 250 mm, with an approximate length of 3 m. These fabrics were deposited onto an LDPE film from the supplied roll. Subsequently, the GF–LDPE sheet was wound and inserted into an aluminum mold with a 300 × 300 mm^2^ cavity.

The compression molding was carried out in a hot press at a temperature of 200 °C and a pressure of 1.6 MPa for 30 min. The molding temperature was selected based on the LDPE’s melting temperature, which is nominally in the range of 105–115 °C. Over-heating is necessary because the mold is initially at room temperature. Furthermore, the long molding time was set to allow for mold pre-heating and laminate consolidation. After molding, the mold was left to cool under pressure, which was released only when the mold reached a temperature of approximately 50–60 °C. The thick composite laminate was extracted by using the removable bottom plate of the mold.

**Figure 4:**
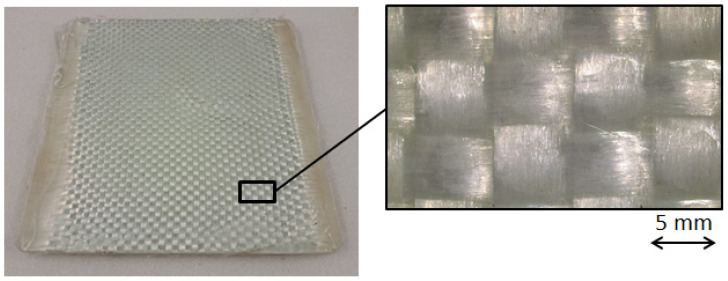
Thick GF–LDPE laminate, created by compression molding.

**Figure 5:**
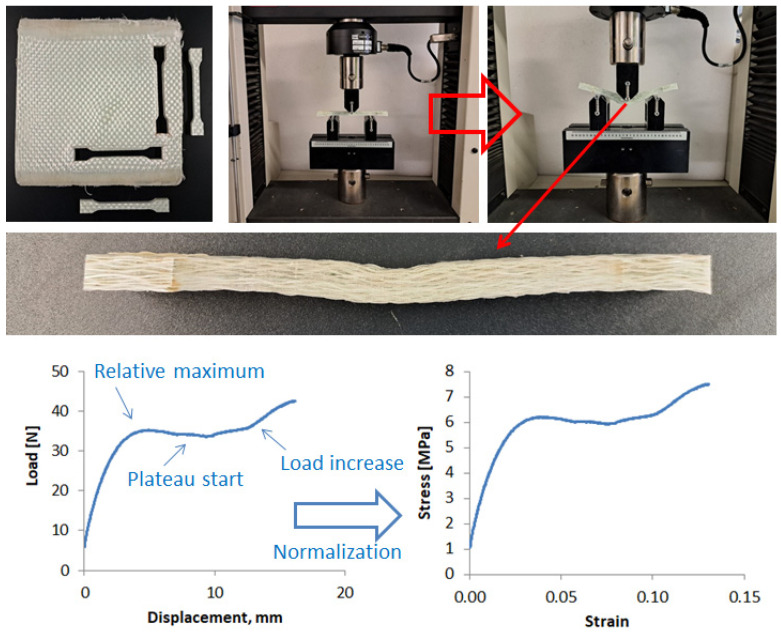
Bending tests on composite samples and typical test curves.

In Figure 4, one molded thick laminate is shown, together with a detail of its surface appearance. Some samples were extracted by shearing for testing. Bending tests were carried out in a universal machine (MTS Insight 5 by MTS Systems, Eden Prairie, MN, USA), according to the ASTM D 790-17 standard [19], at a rate of 10 mm/min with a span length of 80 mm. In Figure 5, the sheared samples are shown before and after testing, together with a typical bending test curve.

The composite samples generally exhibited a tough behavior with low strength and rigidity because of the LDPE matrix. An initial relative maximum of the loading curve was generally present, with a following plateau, as expected. Nevertheless, a load increase was observed after the plateau started because of the thickness of the sample and the contact with the bending supports. For this reason, tests were limited to maximum displacements of 10 mm. However, after load removal, the samples remained clearly deformed, with the evidence of failure in the bending zone.

**Figure 6:**
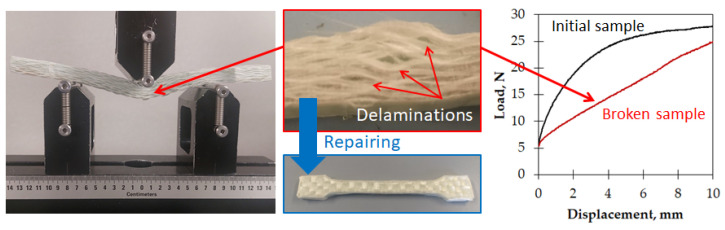
Damage to samples in the repair test.

The thick laminate section was observed by stereoscope (Leica S9i by Leica Microsystems, Wetzlar, Germany) on the sheared samples, and the distance between the plies was estimated, as well as the presence of cavities or other defects. Finally, tests were carried out to estimate the repairing ability of this class of composites. The bending tests were repeated on a sheared sample, similarly to those shown in Figure 5, but at the maximum displacement of 10 mm. Between 2 consecutive tests, a repairing procedure was performed by placing the sample into the same hot press that was already used for the compression molding. In comparison with the manufacturing conditions, the pressure was increased (up to 15 MPa), but the time was strongly reduced (down to 5 min), whereas the plate temperature was left at 200 °C. In Figure 6, the laminate sample is shown under the applied displacement of 10 mm, together with its delamination, its state after unloading, and its appearance after the first repairing operation. To quantify the damage caused by bending, the test was also repeated on the broken sample before the first repairing operation, and the test curves are also shown in Figure 6.

## 3. Results

The thick thermoplastic composite was agglomerated and showed good aesthetics and minimal fiber distortion on most of the molded surfaces (Figure 4).

**Figure 7:**
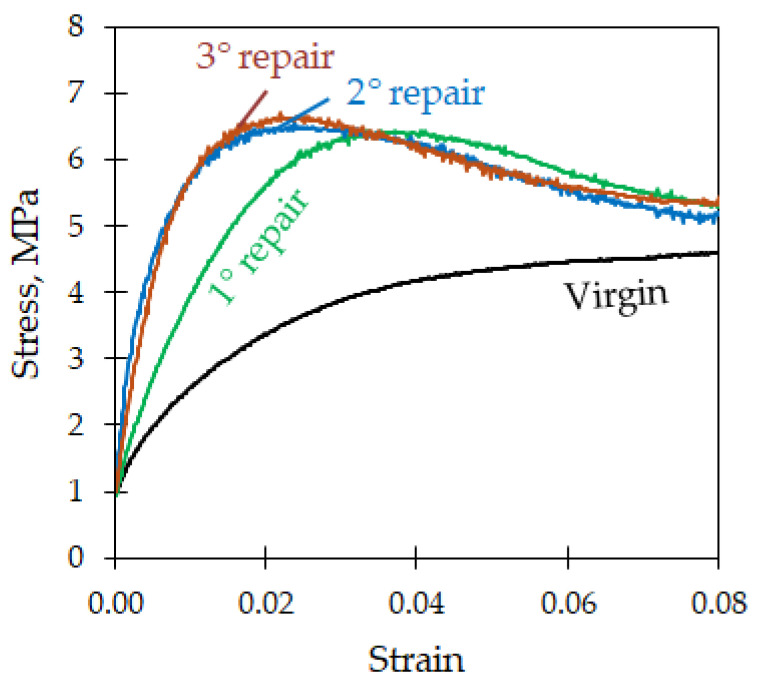
Bending tests on repaired samples in comparison with the virgin.

On the edges, an excess of polymer matrix was present because of the initial clearance between the molding preform and the mold cavity (Figure 3). The final GF–LDPE laminate had a nominal thickness of 9 mm (8.6 ± 0.1 mm, measured on the sheared samples). From the bending tests (Figure 5), an average maximum strength of 6.2 MPa resulted at the strain of 3.70%, with an elastic modulus of 220 MPa. The tests on the repaired samples are shown in Figure 7 after normalization, in comparison with the virgin material. After being repaired, the samples’ performances improved, and the presence of the initial stress maximum became more evident.

**Figure 8:**
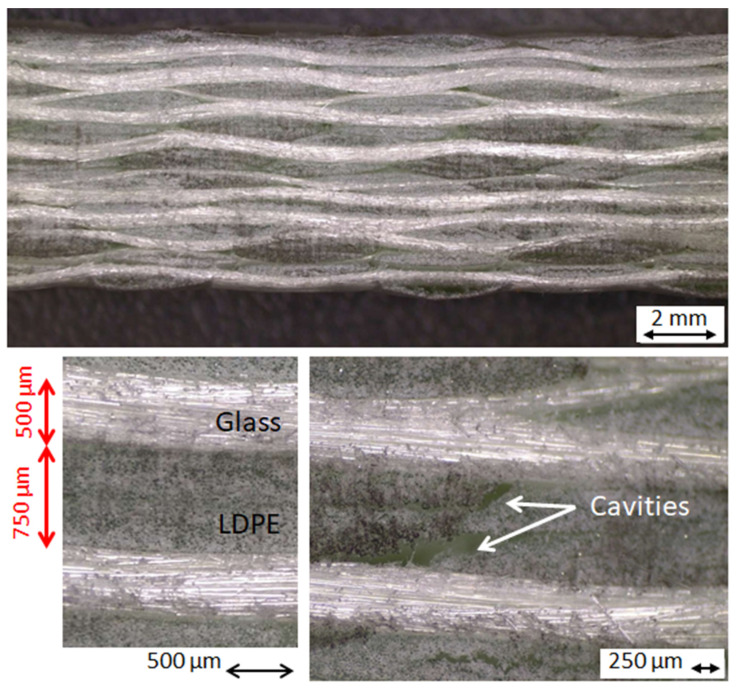
Sections of the sheared sample by a stereoscopic analysis.

In Figure 8, the images of the laminate section, from the sheared sample thickness, are shown: 10 glass-fiber plies can be observed with a thickness of 500 µm, whereas the distance between the plies is up to 750 µm. Some cavities are visible in the matrix between the plies, probably due to shrinking during solidification. In fact, their shape is irregular, and some strands are also visible across. The laminate cross-section was investigated in several points after sample extraction was performed, and all the micrographic images are comparable, thus concluding that a good homogeneity was achieved.

## 4. Discussion

The proposed impregnation procedure seems to be suitable for achieve the good agglomeration of the thick composite. During compression molding, the LDPE film infiltrates through the dry fabrics thanks to its low fluidity, but some cavities are visible in the laminate section (Figure 8). Probably, the application of a vacuum during molding would have reduced their extent. In any case, the cavities have the appearance of shrinking cavities. The cavities partially affected the mechanical performance of the composite as the maximum strength of 6 MPa was measured in the absence of cavities. In the samples where the maximum strength was observed, the maximum elastic modulus of 220 MPa was measured. This fact is not typical of traditional composites, where the strength is related to the presence of cavities and the elastic modulus can be poorly dependent, but it is typical of agglomerated materials. In the presence of many cavities, the strength can reduce by approximately 25%, and the elastic modulus can reduce by over 60%. However, these changes are not dramatic for the thick GF–LDPE composite, which is not characterized by its strength and stiffness but by its toughness, which is always high. An example is given in Figure 7, which initially shows a stress–strain curve that is lower than the sample seen in Figure 5, but the curve rises during the repairing steps and reaches a strength of 6.7 MPa and an elastic modulus of 630 MPa by the end of the third repair, higher than any virgin sample at the first bending test.

Figure 8 shows that 10 glass-fiber plies are visible in the section. The maximum distance between the plies is approximately 750 µm. The average distance between the plies is approximately 400 µm, estimated by considering that the average thickness of the molded laminate is 8.6 mm, and the single reinforcement ply is 500 µm. Therefore, the final composites have a fiber volume content of over 50 vol%.

The mechanical performance of the GF–LDPE composite is generally low as the bending tests are strongly influenced from the matrix, much more so than tensile tests, and the matrix is a very soft polymer, with an elastic modulus in the range of 200–300 MPa. In films, where the mechanical performance increases in relation to the polymer orientation, the elastic modulus reaches 500 MPa with a tensile strength about 10 MPa. In the best case, the laminate samples reached an elastic modulus of over 700 MPa, but these samples were not from the virgin panels. In fact, the highest performances were achieved after the repairing steps. The reason is that the repairing procedure is able to fix the damages that occurred under bending and to reduce the defects from manufacturing, such as low impregnation and the presence of cavities. This improvement was evident because the repairs were performed on a single, small sample and not on the full plate. Nevertheless, the damage which was generated under bending was always severe, as it was possible to observe from the residual deformed shape of the tested samples (Figure 5) or the large delaminations that existed before the repairing step (Figure 6). Thanks to the thermoplastic behavior of the LDPE matrix and its low viscosity, the delaminations were easily closed during repairing, as were the cavities that were shown in Figure 8.

Figure 7 clearly shows the positive effect of the repairing procedure on the performances of the GF–LDPE thick laminate, as well as the strong damage that the sample sustained after the first bending test. By repeating the breaking/repairing cycles, the stress–strain curves approached more closely the expected curve for a fully agglomerated sample, with a relative maximum at low displacements between 6–7 MPa. In the last two repairing steps, the bending curves were almost perfectly superimposed. Therefore, after the initial training, the repairing procedure allowed the sample to reach the nominal material behavior, without the evidence of the previous damage.

**Figure 9:**
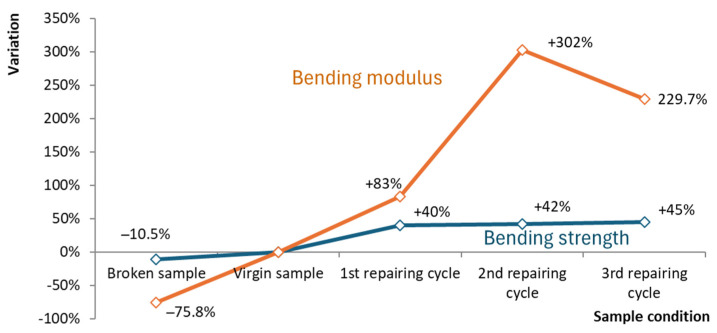
Bending strength and elastic modulus change during the repair test in comparison with the virgin sample.

Figure 9 quantifies this effect by comparing the sample strength and modulus during the different steps with the virgin condition. The bending strength increased after the first repairing step by approximately 40%, from 4.6 MPa to 6.5 MPa, and this increase remained stable also in the following repairing steps, with a maximum of 6.7 MPa by the end of the third repair. The elastic modulus increased to a greater degree than the strength, up to 300%, and did not approach a plateau. Generally, the elastic modulus was more sensitive to damage, as shown by the test of the broken sample, in which the strength reduced by only 11% and the elastic modulus reduced by up to 80%. This difference can be related to the tough behavior of the LDPE matrix, which is generally poorly influenced by the occurrence of delaminations and cavities to a large extent. Glass fibers do not contribute significantly under bending, and the general performances remain low in comparison with traditional fiberglass. Instead, the glass fiber orientation and buckling have a great influence on sample stiffness.

## 5. Conclusions

An easy lamination procedure was designed to allow the recycling of large residual amounts of waste glass-fiber fabrics in the fiberglass industry. Thick thermoplastic composites may be manufactured in parallel with the fiberglass production by recovering other waste materials, such as packaging films or bags for autoclaves. In the current study, a virgin LDPE matrix was chosen for simplicity, and the final composite laminate showed low mechanical properties but very high toughness. However, the main characteristic of this GF–LDPE laminate is its excellent repairability. In fact, the laminate can undergo several breaking/recycling steps without reducing its performance but instead increasing it, as the repairing procedure allowed also for the reduction of defects that occurred during manufacturing. In the end, constant properties were reached in the last repairing step, with the perception of an infinite repairability from bending damages.

**Figure 10:**
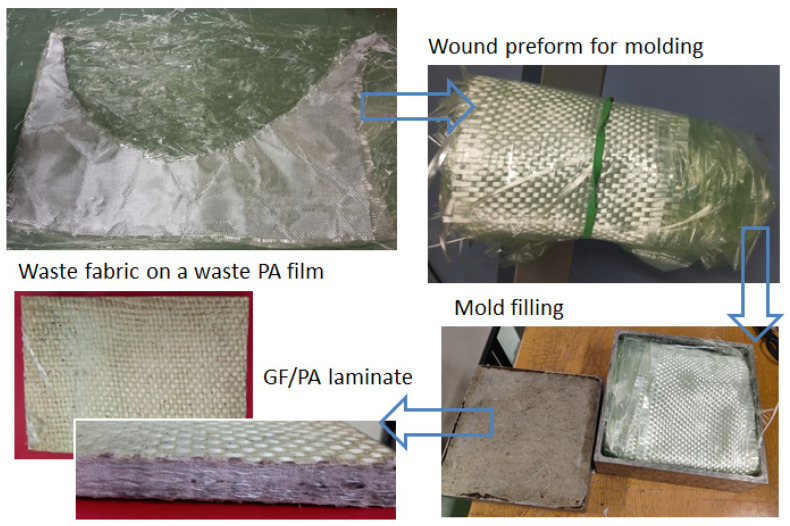
Fabrication of a 100% recycled GF/PA laminate with waste dry glass-fiber fabrics and waste PA films for the vacuum bagging of composites.

The proposed procedure can be extended to many other cases by replacing the virgin LDPE film with other thermoplastics, including thermoplastics from recycling. Different fabrics can be selected as well. An example is given in Figure 10, which shows the same residuals of glass-fiber fabrics being laminated with a waste bag from autoclave molding, made of polyamide (PA).

A good agglomeration was achieved for this filler/matrix combination by compression molding, but higher mechanical performances were reached because of the PA matrix.

The proposed lamination procedure allows for the insertion of each single reinforcement fabric between two sheets of thermoplastic matrix. Fiber impregnation is facilitated, and the amount of matrix can be tailored by selecting the thickness of the thermoplastic sheet or the number of sheets. Many combinations may be explored, and future works will focus on this new class of thick laminates. In general, producing thick thermoplastic composites by compression molding is a complex task because of the difficulty of fiber impregnation and the tendency of the molten matrix to flow away, between the reinforcement plies, instead of passing through the fabrics. The proposed lamination solution seems to be optimal, limiting polymer flow during agglomeration and forcing its penetration between the dry fibers. Some defects can occur, such as cavities, entrapped air, or solidification shrinking, but proper process tuning could minimize them. In the current study, 10-ply glass-fiber-reinforced laminates with a size of 300 × 300 × 10 mm^3^ were molded with excellent repairability. The virgin laminate showed a bending modulus of 205 ± 15 MPa and a bending strength of 5.4 ± 0.8 MPa. The quasi-static properties improved by repairing the single specimen, reaching a bending modulus of over 700 MPa and a bending strength of over 7 MPa.

## Data Availability

Data are contained within the article.
